# Impacts of Air Pollution and Climate Change on Forest Ecosystems — Emerging Research Needs

**DOI:** 10.1100/tsw.2007.52

**Published:** 2007-03-21

**Authors:** Elena Paoletti, Andrzej Bytnerowicz, Chris Andersen, Algirdas Augustaitis, Marco Ferretti, Nancy Grulke, Madeleine S. Günthardt-Goerg, John Innes, Dale Johnson, Dave Karnosky, Jesada Luangjame, Rainer Matyssek, Steven McNulty, Gerhard Müller-Starck, Robert Musselman, Kevin Percy

**Affiliations:** ^1^ Istituto Protezione Piante, Consiglio Nazionale Ricerche, Via Madonna del Piano 10, I-50019 Sesto Fiorentino, Firenze, Italy; ^2^ USDA Forest Service, 4955 Canyon Crest Drive, Riverside, CA 92507, USA; ^3^ USEPA National Health and Environmental Effects Research Lab., Western Ecology Division, 200 SW 35th St., Corvallis, OR 97333, USA; ^4^ Forest Monitoring Laboratory, Lithuanian University of Agriculture, Kaunas 4324, Lithuania; ^5^ Linnæambiente Ricerca Applicata Srl, Via G. Sirtori 37, I-50137 Firenze, Italy; ^6^ Swiss Federal Institute for Forest, Snow and Landscape Research, Zürcherstrasse 111, CH-8903 Birmensdorf, Switzerland; ^7^ Department of Forest Resources Management, Faculty of Forestry, The University of British Columbia, 2^nd^ Floor, Forest Sciences Centre #2045-2424 Main Mall, Vancouver BC V6T 1Z4, Canada; ^8^ Environmental and Resource Sciences, Fleischmann Agriculture, MS 370, University of Nevada, Reno, NV 89557, USA; ^9^ School of Forest Resources and Environmental Science, Michigan Technological University, 101 U.J. Noblet Forestry Building, 1400 Townsend Drive, Houghton, MI 49931-1295, USA; ^10^ Royal Forest Department, Silvicultural Research Division, 61 Phahlythin Road, Bangkok 10900, Thailand; ^11^ Technische Universität München, Department of Ecology/Ecophysiology of Plants, Am Hochanger 13, D-85354 Freising, Germany; ^12^ USDA Forest Service, Southern Global Change Program, Venture Center II, Suite 300, Raleigh, NC 27606, USA; ^13^ Technische Universität München, Fachgebiet Forstgenetik, Am Hochanger 13, D-85354 Freising, Germany; ^14^ USDA Forest Service, 240 West Prospect Road, Fort Collins, CO 80526, USA; ^15^ Natural Resources Canada, Canadian Forest Service, 1350 Regent Street, Fredericton NB E3B 5P7, Canada

**Keywords:** Air Pollutants, Forests, Global Change, State of the Art

## Abstract

Outcomes from the 22^nd^ meeting for Specialists in Air Pollution Effects on Forest Ecosystems “Forests under Anthropogenic Pressure Effects of Air Pollution, Climate Change and Urban Development”, September 1016, 2006, Riverside, CA, are summarized. Tropospheric or ground-level ozone (O_3_) is still the phytotoxic air pollutant of major interest. Challenging issues are how to make O_3_ standards or critical levels more biologically based and at the same time practical for wide use; quantification of plant detoxification processes in flux modeling; inclusion of multiple environmental stresses in critical load determinations; new concept development for nitrogen saturation; interactions between air pollution, climate, and forest pests; effects of forest fire on air quality; the capacity of forests to sequester carbon under changing climatic conditions and coexposure to elevated levels of air pollutants; enhanced linkage between molecular biology, biochemistry, physiology, and morphological traits.

## INTRODUCTION

The International Union of Forest Research Organizations (IUFRO) 22nd biennial meeting for Specialists in Air Pollution Effects on Forest Ecosystems "Forests under Anthropogenic Pressure – Effects of Air Pollution, Climate Change and Urban Development" was held September 10–16, 2006, in Riverside, CA. The meeting was hosted by the USDA Forest Service, Pacific Southwest Research Station, on behalf of IUFRO Research Group 7.01.00 "Impacts of Air Pollution and Climate Change on Forest Ecosystems". A satellite symposium on "Forest Fires and Air Pollution Issues" was also held. This report highlights new findings and outlines research needs (Table 1) related to the topics from the two meetings. It is not intended to serve as a summary of all the papers presented in the meetings; rather, the reader is referred to the list of abstracts and proceedings[1] for the latter.

## AIR POLLUTION AND FOREST FIRES

The recent increase in application of fire as a tool for land-use change has resulted in more frequent occurrences of extended fire and smoke episodes worldwide with serious consequences for human and ecosystem health. Some of these events have been associated with droughts that may be attributed to increasing regional climate variability. International efforts are needed to address the underlying causes and avoidance of excessive fire application, and to implement sound fire and smoke management practices.

Fires, both wild and prescribed, can significantly contribute to ambient air pollution. Consequently, air quality concerns and regulations are major constraints to the implementation of prescribed fire strategies in many instances. Modeling is an important tool for characterizing the transport of polluted air masses resulting from forest fires, and for predicting forest fire effects on air quality. Comparisons between air pollutant concentrations estimated with and without forest fires may allow us to assess forest fire contribution to ambient air pollution. Consequently, there is an urgent need to develop predictive models to assist land and air resource managers in the timely and controlled use of fires that would not cause exceedances of criteria air pollutant standards (e.g., particulate matter [$PM_{2.5}$ and $PM_{10}$] and $O_3$).

Fire has emerged as a major issue in forests with Mediterranean-type climates where incidence, duration, and severity of fires have significantly increased. Climate warming may be contributing to this increase. For example, in the U.S., the combination of 90 years of intensive fire suppression and significantly warmer air temperatures since the 1990s has led to successive years of record fire occurrence. Although the ecological changes in forests caused by wildfire are profound and generally well known, their effects on ecosystems may be more pronounced and long lasting in regions with warming climate. The potential for prescribed fire to mitigate nitrogen (N) saturation resulting from atmospheric deposition is of particular interest. There are only few other disturbances that can cause comparable N loss in such a short amount of time. The possibility for prescribed fire to mitigate N-saturated sites needs to be explored with field experiments. However, there is also a clear need to understand the fate of released N gases and aerosols, and their potential effects on air quality and N deposition downwind from the fires. Furthermore, fire can change base cations from organic compounds to oxides and hydroxides in ash that, in turn, become readily available for replenishment of base cations on exchange sites, increasing soil short-term acid buffering capacity. Increases in soil pH are commonly observed following fire.

**TABLE 1**
**Emerging Research Issues at the 22nd Meeting for Specialists in Air Pollution Effects on Forest Ecosystems**

| Subject | Challenging Issues |
|---|---|
| $O_3$ | Understanding the disruption of metabolic pathways (ascorbate and ethylene) |
| | Quantification of plant detoxification processes in the flux models |
| | Quantification of stomatal and nonstomatal $O_3$ fluxes in many plant species |
| | Translating stomatal $O_3$ flux to biological effects |
| | Using long-term experiments that utilize free-air $O_3$ release systems |
| | Making $O_3$ standards more biologically based, statistically sound, yet practical for wide use by air quality regulators |
| Nitrogen | Developing a new concept of nitrogen saturation |
| | Inclusion of multiple environmental stresses in critical load determinations |
| Elevated $CO_2$ | Understanding capacity of forests to sequester carbon under changing climatic conditions and coexposure to elevated levels of pollution |
| | Using long-term experiments that utilize free-air $CO_2$ and $O_3$ release systems |
| Air pollution | Characterizing effects of forest fire on air quality |
| | Understanding effects of secondary organic aerosols on trees |
| | Linking molecular biology, biochemistry, physiology, and morphological traits |
| | Understanding environment-dependent impacts of gene regulation on the expression of traits that are important for adaptation and survival |
| | Understanding effects of plant competitiveness (monocultures vs. mixed cultures, single trees vs. community responses) on plant responses to stressors |
| | Establishing mechanisms of whole-tree allocation (including genetic control) under climate change scenarios |
| | Characterizing effects of air pollution on physiological and allocation responses of forest ecosystems |
| | Understanding how nutrient availability limits long-term carbon sequestration |
| | Understanding how air pollution affects hydrological and soil processes |
| Interactions | Establishing interactive effects of air pollution and climate change, in particular drought |
| | Understanding impacts of air pollution on soil fauna, phyllosphere biota, and biogenic emissions |
| | Establishing trophic, competitive, and host/parasite relationships under changing pollution and climate regimes, including mycorrhizas and forest pests |
| | Characterizing impacts of pollution and climate change on soil carbon dynamics (in particular root production and turnover in natural ecosystems) |
| | Understanding effects of climate change and air pollution on plant phenology and reproductive fitness |
| Risk assessment | Developing scientifically defensible risk assessments supported by appropriate endpoints and exposure- or dose-response models |
| | Improving communication between scientists and policy makers |

The increase in forest susceptibility to fires caused by air pollution is also of interest. Elevated background levels of $O_3$ and N deposition increase leaf turnover rates, as well as N and lignin content in needles. This reduces litter decomposition and increases fuel buildup. Both $O_3$ exposure and elevated available N may reduce root/shoot mass and plant control of water loss, thus increasing tree susceptibility to drought stress and fire. Better understanding of mechanisms of air pollution effects on wild-land fires in forests and other terrestrial ecosystems is needed.

The sources of forest fire emissions and effects of forest fires on carbon budget should be quantified. Improved understanding of the effects of changing fire regimes on all aspects of the carbon cycle is needed before we can better predict the magnitude, or even the direction, of the effect of changing fire regimes on the global carbon budget.

## AIR POLLUTION AND CLIMATE CHANGE

Research on air pollution effects on forests has often been focused on the impacts of air pollutants on trees. However, forest ecosystem health encompasses much greater complexity than simply tree health. The potential for air pollution effects on biogenic VOC emissions, phyllosphere biota, interorganismic interactions, and soil fauna must also be considered when assessing forest health. The impacts of air pollution and climate change on forest ecosystems have been traditionally studied and reported on separately. Many air pollutants and greenhouse gases ($O_3$ is both) have common sources, contribute to global warming through increased radiative forcing, interact in the atmosphere, and jointly affect ecosystems. Their combined effects may significantly differ from a sum of separate effects. Some pollutants that are now of emerging concern may be of significant importance for forest health. These include, in particular, secondary organic aerosols, especially aerosol deposition rates to forests and mechanisms for increasing leaf wetness.

The technical facilities to test air pollution effects on plant systems have been developed: greenhouses, climate chambers, open-top chambers, dynamic leaf cuvettes, branch and single tree chambers, EDU application, free-air fumigation systems, and sapflow measurements. Sapflow can be a useful tool to validate stomatal functions and to assess whole-plant $O_3$ uptake, even though time offsets may exist between sapflow and meteorological parameters. There has been a significant effort to move away from chamber studies to field studies in forest health indicator development, which has improved our understanding of the scope and extent of air pollution effects in forested ecosystems. Together with innovative technical facilities for pollutant exposure or measurement, new tools for data analysis and presentation including dynamic general vegetation models, meta-analysis, and redundancy canonical analysis are now available.

Presentations were dedicated to elevated carbon dioxide ($CO_2$), N deposition and availability, climatic factors, pathogens, acidification, heavy metals, and especially to $O_3$. More than a third of the 80 oral and poster presentations were on $O_3$ effects, which remains the phytotoxic pollutant of most concern.

There was much discussion about $O_3$ critical levels in an attempt to improve regulatory standards. Both North Americans and Europeans use exposure-based metrics, while the Europeans are moving toward the use of flux-based metrics for $O_3$, with the latter gaining momentum. The definition of critical levels has changed over the years to allow for the incorporation of the flux concept. A key component of flux-based critical levels is knowledge about the receptor including growth, yield, visible injury, and seed production. One of the most pressing challenges in setting an $O_3$ standard involves our ability to make $O_3$ critical levels more mechanistic by quantifying both stomatal $O_3$ flux and the plant's defense capacity. Flux alone is not sufficient, because plant response depends on nonlinear mechanisms, such as defense, ontogeny, and phenology. Practical application of a flux-based risk assessment requires estimates of these parameters that are species specific. However, data for parameterization of flux-based critical level models are missing for most plant species. An interesting contribution was presented for the Mediterranean forest trees. Results from leaf-level or canopy-level measurements showed significant deviations from the currently used flux model, which has implications for $O_3$ flux calculations. These results demand adequate validation of models before they are used to predict productivity losses. Free-air $O_3$ release systems at field sites can provide information on underlying mechanisms.

To understand the integrated effects of air pollution and climate change on forest ecosystems, a multifactorial and cause/effect-related approach is required. Experiments that focused on more than one stress factor simultaneously (e.g., reporting on complex responses in cases of interactions between air pollution, climate, and forest pests) resulted in novel results that will have wider application in risk assessment. Examples were provided of conflicting plant responses that do not only depend on the given pollutant and pathogen or herbivorous insect, but also depend on the plant species or even genotype and the specific growth conditions. Interactions between pathogens and abiotic factors in plant response demonstrate the need for increased awareness of these complexities, as they affect competition, species composition, and ecosystem functioning. Subtle biotic interactions within a forest may have more important consequences than the direct effects of pollution. Current knowledge on this issue leads to the assumption that chronic pollutant impacts may weaken plant defense to other stress factors rather than to strengthen it. Such a pathway of pollutant action is believed to be one cause of the successional changes in the $O_3$-impacted forests of California. Long-term changes in the gene pool of tree populations may result in altered species composition with consequences for food webs and fire ecology. Prognoses become uncertain when inter- and intra tree species competition is more important than stress by pollutants and parasites. Pathogen infection may be more sensitive to climate than pollutants. Apparently, ecological scenarios vary in regard to plant and parasite responses, and difficulties in a full understanding will prevail as long as mechanistic explanation, including genetic control, is inadequate.

Guidance for new investigations can be provided by theories about resource allocation such as the Growth Differentiation Balance Theory (GDB)[2]. GDB addresses the trade-off between growth- and defense-related metabolism, with the latter being favored at low resource availability, and the former favored at high resource availability. Metabolic control and metabolic activity (i.e., molecular and "organismic" biology) should be studied in parallel; the latter representing the biochemical and ecophysiological process levels of the whole plant. The trade-off addressed by GDB needs to consider also the resource exchange with other pools, such as the mycorrhizosphere. In addition, conventional scaling of processes needs to account for ontogenetic stages and growth conditions (controlled vs. field), with actual pollutant uptake and effective dose having paramount importance. Research outcome needs to validate models as an analytical and integrative tool capable in quantifying allocation trade-offs across scenarios. The ultimate goal is the mechanistic understanding of interactions, implying spatiotemporal integration of resource allocation across the plant-plant, plant-parasite, and plant-mycorrhizosphere relationships, while examining findings for their extent of generality across site conditions, tree life forms, and ontogenetic stages.

## Detection, Monitoring, and Evaluation

Monitoring is essential to document current status and changes in forest health. Two topics that came to light during the meeting merit further investigations: (1) changes in plant phenology due to climate change and (2) plant competitive response to pollution. The length of the growing season is an important factor affecting forest productivity. Compared to historic conditions, the growing season now begins up to 15 days earlier in central-northern Europe. At the community level, species-rich plant communities were shown to experience more disturbance impacts under pollution compared to species-poor communities. This conclusion contradicts the widely accepted theory of greater resilience of species-rich communities and contributes to the discussion on the relationships between diversity and stability.

As in the previous meetings, $O_3$ was the pollutant of major concern for forest scientists and this reflects the activity and the large amount of work carried out at the international level. Attempts to study $O_3$ effects on forests were presented including statistical (i.e., correlative) approaches and perspectives on risk assessment. Under low $O_3$ levels, peak concentrations of $O_3$ seemed to have more significant effects on tree defoliation and increment rather than mean or integrated AOT40 values. However, $O_3$ concentrations and exposure-based indices explained a limited portion of the total variance in crown transparency and basal area increment. The flux-based approach in risk assessment was advocated. Many questions remain unanswered including: (1) Is nonstomatal flux an important part of total $O_3$ flux? and (2) Can cumulated flux and cumulated exposure diverge considerably according to the precipitation regime? There is a clear need for coupled long-term monitoring and short-term experiments to translate stomatal $O_3$ flux to biological effects. In this context, long-term studies may provide valuable information. In particular, a better integration among related programs (e.g., ICP Integrated Monitoring, ICP Forests, and national projects in Europe) is needed.

## Mechanisms of Action and Indicator Development

Mechanisms of action and indicators of pollution stress continue to be areas of the largest contributions to air pollution research. It is particularly challenging to identify specific or unique indicators for individual stresses. Many biochemical indicators are not pollutant specific, making it difficult to identify causative agents in atmospheres with a mixture of pollutants. Detailed microscopic techniques are being used successfully for diagnosing $O_3$ and other stresses, such as heavy metals, irrespective of the site and species, although certain species-specific responses exist.

Over the last decade, widespread use of stable isotope technology in air pollution research has improved our understanding of the physiological effects of pollutant stress and, in some cases, has been used to reveal pollutant sources. The increased availability of mass spectrometers has driven the development and application of this powerful analytical tool. There is no doubt that stable isotope technology will be an important component of future air pollution research, particularly to assess effects in the field.

Modeling stomatal behavior continues to be a significant challenge as we attempt to move away from exposure statistics and replace them with a more meaningful estimate of actual uptake or dose. Most models and exposure statistics consider only daylight hours when calculating $O_3$ exposure and/or uptake, yet new results show that some species exhibit stomatal opening at night. In some cases, stomata remain open at night after daytime $O_3$ exposure. These responses appear to be transient and reversible, and may lead to sluggish stomatal behavior. In addition, the presence of $O_3$ may influence the response of stomata to light, further complicating estimates of stomatal uptake. Models need to include moisture stress as well as VPD when estimating uptake, and recent efforts to use eddy-flux estimates may lead to improved estimates of $O_3$ uptake. Collectively, these efforts will provide new information that may be important to establish protective air quality standards in North America and critical levels in Europe.

Physiological understanding of plant response to $O_3$ has been studied for decades, yet there is still much that is not understood. The perception of visual leaf injury, night and winter exposure effects, climate and interorganismic interactions, acclimation and adaptation potential of natural vegetation, and the disruption of metabolic pathways are all largely unknown. With the development of molecular tools, such as microarrays, researchers now have the ability to look at changes in gene expression and to examine how stresses, such as $O_3$, may influence plant metabolism at the molecular level. These powerful tools will continue to provide important mechanistic information on plant response to $O_3$ and other atmospheric stresses, and may yield stress-specific indicators of response. However, integration is needed across the molecular and biochemical/(eco-)physiological process levels to develop a mechanistic understanding at the whole-plant level.

It is clear that plant response to multiple stresses and multiple pollutant interactions is one of the most significant challenges facing air pollution researchers. Ozone during the summer has been shown to affect the ability of certain tree species to tolerate freeze-thaw events. Because of the dynamic nature and timing of specific climatic events that lead to this response, it may be difficult to link resulting damage to $O_3$. It was also reported that drought and $O_3$ interact in certain tree species. Again, these responses are species specific, site specific, and climatic-zone specific, presenting a significant challenge for unifying an approach to assessing forest $O_3$ flux- (Europe) or exposure-based (North America) effects.

## Soils and Nutrient Cycling

Two topics that were important during the meeting merit continued consideration: the concepts of N saturation and critical loads. Many definitions of N saturation have been offered including some based on the degree of net retention of N deposited to ecosystems. Results from the N application at the Fernow Experimental Forest in West Virginia bring this into focus: while total N retention went up to ~90% with N

amendments (suggesting less N saturation), soil solution nitrate levels increased (suggesting more N saturation). Thus, it does not seem advisable to define N saturation on the basis of net ecosystem N retention. Other presentations showed that critical loads for N vary substantially with other conditions, such as drought, levels of other pollutants, and stand age, bringing into question the concept of a single critical load for an entire region. An update on the development of critical loads estimates for N, sulfur, and acidity for U.S. forest ecosystems, and a case study where critical loads for N were dependent on multiple stresses (including drought and insects) suggest that multiple stresses must be included in critical loads analyses.

Although long considered a negative effect, recent studies have shown that soil compaction in coarse-textured soils on drought sites can cause greater plant growth by increasing soil water-holding capacity. Similar to many other factors in soil fertility, it is the degree of compaction and the site conditions that determine its effects, and broad generalizations are not advisable. A second new area of soil science that relates to many air pollution issues is that of “hot spots” and “hot moments”, about which several papers have been recently written. These are microsite locations in the soil where nutrient availability is high (hot spots), from which plant roots can take up nutrients that are limiting in the bulk soil. These hot spots are ephemeral, as plant roots deplete them during “hot moments”. As these sites move from place to place, plants roots also concentrate in these microsite locations, thus potentially allowing plants to obtain a larger proportion of soil nutrients than may be indicated from bulk soil analyses. This is an area that can change how we view plant root–microbial competition for N and other nutrients, and deserves much more research and consideration.

### Genetic Aspects

The development of molecular tools that address genomics, transcriptomics, proteomics, and metabolomics is rapidly expanding our understanding of the basic genetic control of physiological traits. These approaches are complemented by the now complete genome sequences for *Populus*. Together with QTL (Quantitative Trait Locus) approaches, researchers have unprecedented opportunities to link variation in complex physiological traits to DNA sequences and to specific chromosomes. This new approach is particularly useful to examine the responses of trees to stresses, such as air pollution and climate change. Studies on DNA methylation demonstrate impacts of environmental conditions on gene regulation and corresponding modifications of the adaptive abilities of juvenile plants of *Picea abies* to climatic stresses.

Another emerging research topic presented at the meeting was the examination of reproductive fitness of trees exposed for their entire life history to elevated levels of $CO_2$ and $O_3$. Until recently, this type of research with long-lived trees, which often have juvenile periods of 5–20 years or longer, was impossible. The development of free-air $CO_2$ and $O_3$ exposure (FACE) systems is now allowing for such research to be conducted. This research will help to predict community dynamics for forest ecosystems exposed to rising atmospheric concentrations of greenhouse gases.

## CONCLUSIONS

Field studies, monitoring, and modeling work were presented at the 22$^{nd}$ IUFRO Meeting for Specialists in Air Pollution Effects on Forest Ecosystems, documenting air pollution and climate change impacts on forests in Europe and North America. Although many questions remain, we now know a great deal more about how air pollution directly and indirectly affects individual tree species. However, forest ecosystems are much more complex and difficult to model. In addition, as air pollution increases and the climate changes, the threats to forest health from air pollution will also change. Long-term experiments utilizing free-air $CO_2$/$O_3$ release systems are the latest and currently best approach for understanding the cause/effect-based relationships between pollutants and other stresses at the stand and ecosystem level.

The meeting identified a number of emerging research needs including understanding the disruption of metabolic pathways (ascorbate and ethylene); impacts of air pollution on soil fauna, phyllosphere biota, insect interactions, and biogenic emissions; effects of secondary organic aerosols on trees; the effects dependant on plant competitiveness (monocultures vs. mixed cultures, single trees vs. community responses); effects of climate change and air pollution on plant phenology and reproductive fitness; mechanisms of whole-tree allocation (including genetic control) under ecologically meaningful climate change scenarios to develop reliable risk assessments; how to make $O_3$ standards more biologically based and at the same time practical for a wide use; effects of forest fire on air quality; the concepts of N saturation and critical loads; and trophic, competitive, and host/parasite relationships under changing pollution and climate regimes, including the mycorrhizosphere.

Interactions between air pollution, climate, and forest pests are still an issue of ongoing debate, as it has become a widespread opinion that pollutants may predispose trees to injury induced by other kinds of stress, eventually leading to decline. Little information is available about environment-dependent impacts of gene regulation on the expression of traits that are important for adaptation and survival. Little is still known about the capacity of forests to sequester carbon under real-world conditions (i.e., under changing climatic conditions coupled with coexposure to elevated levels of pollution). This knowledge is needed to predict how the carbon sequestration potential of the world's forests may change in the future, which includes the interactions between air pollution and drought, how air pollution affects physiological and allocation responses of forest ecosystems, how nutrient availability limits long-term carbon sequestration, and how air pollution affects hydrological and soil processes. The impacts of pollution and climate change on soil carbon dynamics are poorly understood, in part due to a lack of information regarding root production and turnover in natural ecosystems. We must promote management of forests based on good science to maximize their carbon retention.

There is a clear need of improved communication between scientists and policy makers to achieve a science-based management and address future research and environmental policies. Global issues are also fundamentally a conglomeration of local issues that require a coordinated international effort. Particular focus should be placed on interdisciplinary research. IUFRO promotes global cooperation in forest-related research and is the best site for scientific knowledge dissemination and international policy development. A new IUFRO Working Party (7.01.05) has been established within Research Group 7.01.00 with the expressed intention of integrating the work of the other four Working Parties through modeling, leading to enhanced risk assessment and integration of multidisciplinary knowledge about air pollution and climate change impacts on forests.

## ACKNOWLEDGMENTS

We wish to thank the USDA Forest Service, Pacific Southwest Research Station, for kindly hosting this conference; speakers and participants, for the quality of their contributions and for the fruitful discussions.
